# Supplementary material for: Circular RNA encoded MET variant promotes glioblastoma tumorigenesis
Source: Nat Commun. 2023 Jul 25;14:4467. doi: 10.1038/s41467-023-40212-1 (PMC10368723; doi:10.1038/s41467-023-40212-1)
Supplement: Supplementary file 1 — Supplementary Information [file 41467_2023_40212_MOESM1_ESM.pdf]

Supplementary Information

Circular RNA Encoded MET Variant Promotes Glioblastoma Tumorigenesis

Jian Zhong, Xujia Wu, Yixin Gao, Junju Chen, Maolei Zhang, Huangkai Zhou, Jia Yang, Feizhe Xiao, Xuesong Yang, Nunu Huang, Haoyue Qi, Xiuxing Wang, Fan Bai, Yu Shi, Nu Zhang

Supplementary Figure 1

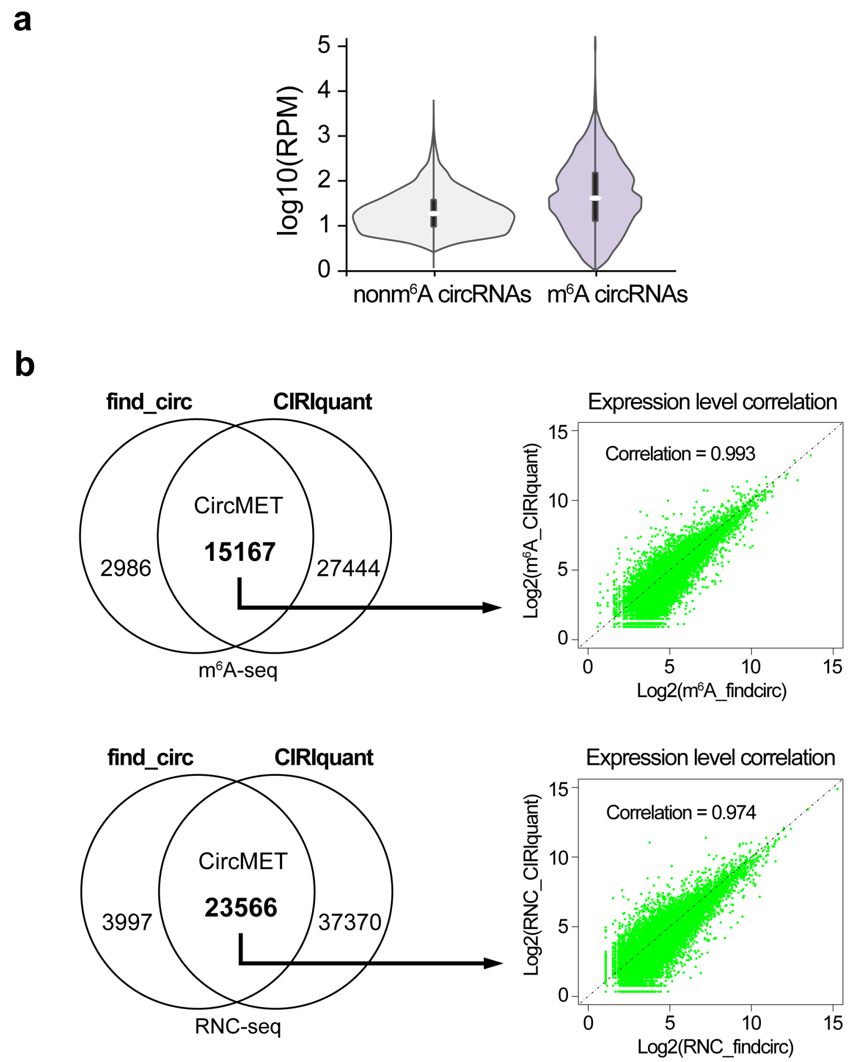

Supplementary Figure 1. M^{6}A-seq of human glioma tissue; comparison of circRNA-identifying algorithms (related to Fig. 1)

a. Violin plot illustrating the expression level of m^{6}A circRNAs (n=1425) and non m^{6}A circRNAs (n=16728) in the 10 human glioma samples. The thick vertical lines indicate the interquartile range. The thick white horizontal lines indicate the median. The whiskers indicate the maxima and minima. Two-sided unpaired Student's *t* test, *P*=0.019.

b. Left, Venn diagram showing circRNAs identified by both algorithms (including circMET) using m^{6}A-seq or RNC-seq data. Right, correlation of the expression levels of circRNAs identified by both algorithms.

Source data are provided in the Supplementary Data.

Supplementary Figure 2

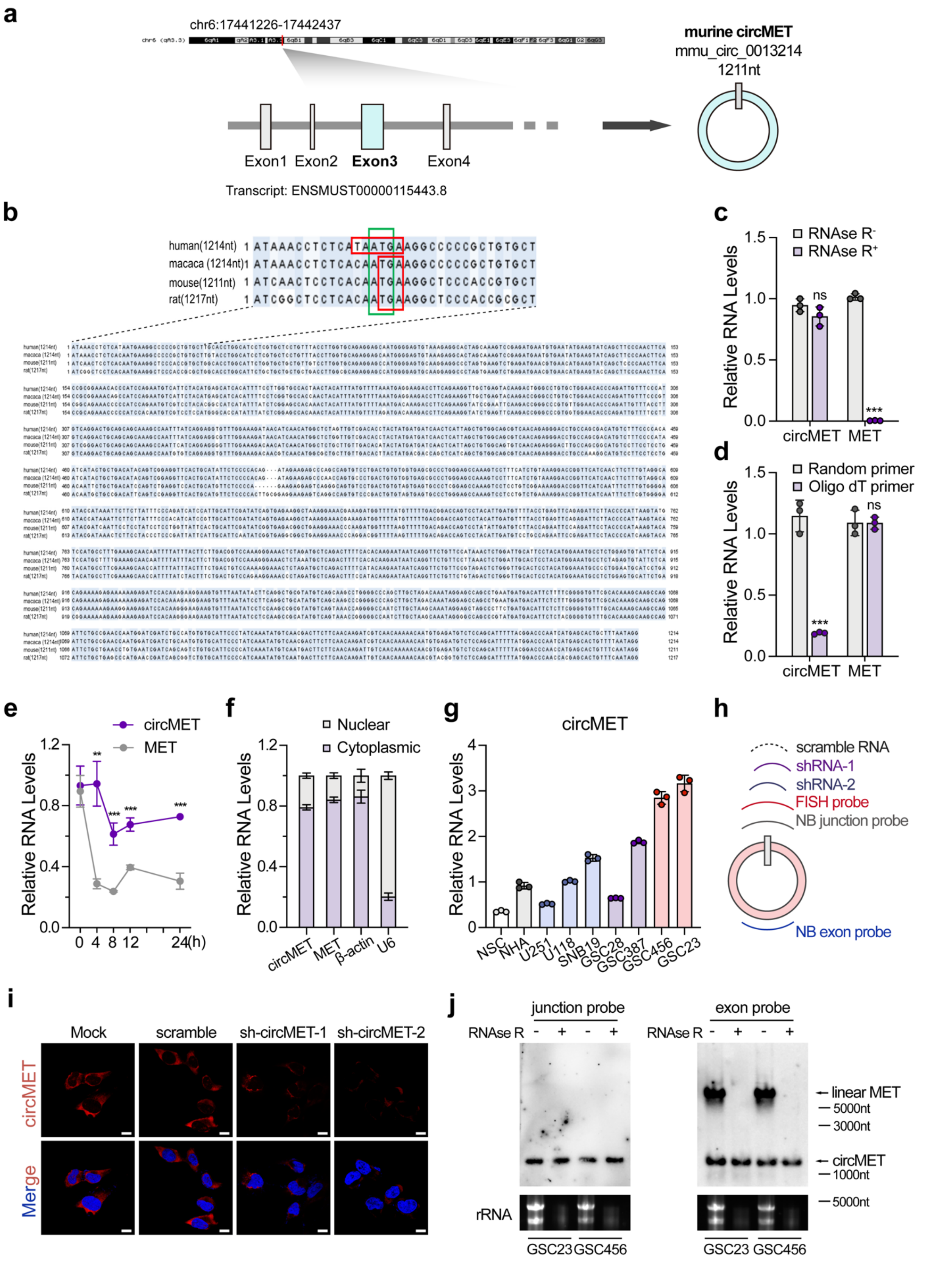

Supplementary Figure 2. Illustration of murine circMET; characteristics of human circMET. (Related to Fig. 2)

a. Illustration of the annotated genomic region of murine circMET. Exon 3 of murine MET pre-mRNA is back-spliced into murine circMET (mmu_circ_0013214).

b. Comparison of circMET sequences among humans, Macaca, mice and rats. Green frame, start codon; red frame, stop codon.

c. Relative RNA levels of circMET and MET in GSC23 cells with or without RNase R treatment. n=3 independent experiments. MET, P=2.97e-07.

d. Oligo-dT or random primers were used to reverse-transcribe total RNA from GSC23 cells. The relative RNA levels of circMET and MET were measured by qPCR. n=3 independent experiments. CircMET, P=2.17e-04.

e. Half-lives of circMET and MET mRNA after treatment with actinomycin D (2 μg/ml) in GSC23 cells. n=3 independent experiments. 4h, P=0.0016; 8h, P=8.70e-04; 12h, P=4.60e-04; 24h, P=1.72e-04.

f. The RNA levels of circMET and MET in the cytoplasmic and nuclear fractions of GSC23 cells. β-actin and U6 were used as the cytoplasmic and nuclear controls, respectively. n=3 independent experiments.

g. Relative RNA levels of circMET in NSC, NHA, GBM cell lines (U251, U118 and SNB19) and GSC cell lines. n=3 independent experiments.

h. Illustration of shRNAs, FISH and Northern blot probes specific for the junction site of circMET. A Northern blot exon probe recognizing both circMET and linear MET was also designed.

i. Representative images of circMET in GSCs detected by FISH. CircMET shRNAs were applied to verify the specificity of the FISH probes. Representative of three independent experiments. Scale bar, 10 μm.

j. Northern blots using junction probes (left) or exon probes (right) in the presence or absence of RNase R treatment in GSC456 and GSC23. Representative of three independent experiments.

The data are presented as the mean±SD. Unpaired two-sided Student's *t* test in **c-e**. Source data are provided as a Source Data file.

Supplementary Figure 3

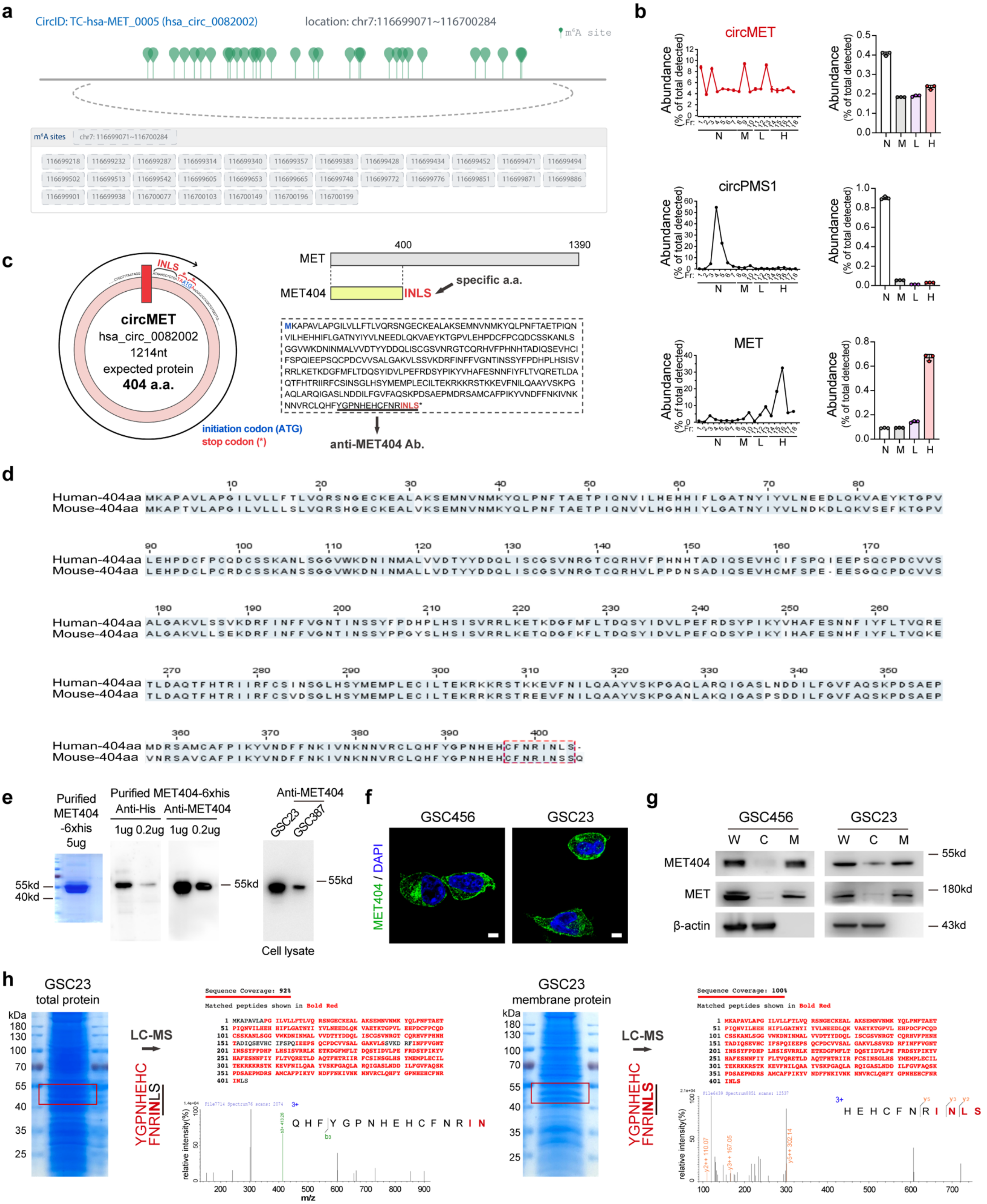

Supplementary Figure 3. Characteristics of MET404 (related to Fig. 2)

a. Illustration of m^6A-modified sites on circMET (hsa_circ_09226) adapted from the TransCirc database [https://www.biosino.org/transcirc/search].

b. Polysome profiling of circMET. The circMET levels of the indicated fractions were measured by qPCR. CircPMS1 and MET served as negative and positive controls, respectively. N, nonribosome; M, monosome; L, light polysome; H, heavy polysome. n=3 independent experiments. The data are presented as the mean±SD.

c. Left, illustration of the circMET ORF with the unique C-terminus consisting of four amino acids (INLS). Right, comparison of amino acid sequences between MET404 and MET. The full peptide sequence of MET404 is shown inside the dashed box. A custom antibody against the indicated unique C-terminus was designed.

d. Comparison of the MET404 peptide sequence between humans and mice.

e. Left, Coomassie blue-stained purified MET404 with a C-terminal His tag. Middle, immunoblot of purified MET404 using an anti-His antibody and the custom anti-MET404 antibody. Right, immunoblot of MET404 in GSC23/GSC387. Representative of three independent experiments.

f. Representative immunofluorescence images of GSC456 and GSC23 stained with anti-MET404 antibody. Scale bar, 5 μm. Representative of three independent experiments.

g. Immunoblotting for MET404, the membrane marker MET and the cytoplasmic marker β-actin in membrane/cytoplasmic fractions of GSC456 and GSC23 cells. Representative of three independent experiments. W, whole-cell lysate; C, cytoplasm; M, membrane.

h. Coomassie blue stained total proteins (left) or membrane proteins (right) of GSC23 cells. Proteins of the indicated molecular weight were collected for LC–MS analysis. The sequence coverage and detection of the unique C-terminal amino acids are shown to the right of the stained gel. Representative of three independent experiments.

Source data are provided as a Source Data file.

Supplementary Figure 4

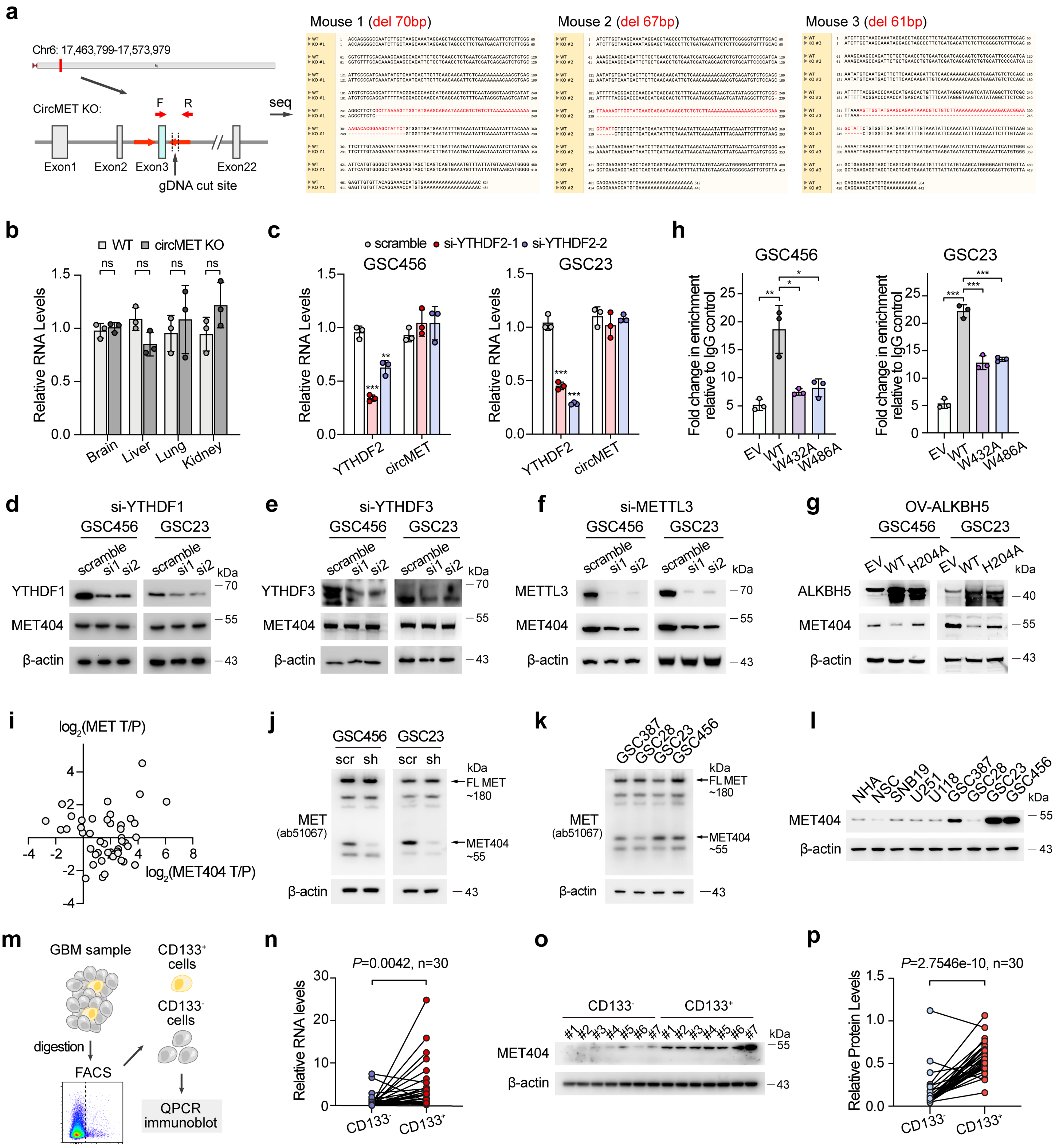

Supplementary Figure 4. CircMET-encoded MET404 is driven by the m^{6}A reader YTHDF2 and is highly expressed in GSCs (related to Fig. 2)

a. Left, illustration of the gDNA cut site in the flanking intron containing orientation-opposite complementary sequences of mouse circMET. Right, representative alignments showing successful deletion of the targeted sequences in the circMET KO mouse genome.

b. MET RNA levels in the indicated organs of the circMET KO mouse model. n=3 independent experiments.

c. Relative RNA levels of YTHDF2 and circMET in GSC456/GSC23 cells transfected with YTHDF2 siRNAs. n=3 independent experiments. GSC456, scr vs si-1, *P*=8.26e-05; scr vs si-2, *P*=0.0030. GSC23, scr vs si-1, *P*=2.09e-04; scr vs si-2, *P*=5.33e-05.

d. Protein levels of MET404 in GSC456/GSC23 cells transfected with YTHDF1 siRNAs.

e. Protein levels of MET404 in GSC456/GSC23 cells transfected with YTHDF3 siRNAs.

f. Protein levels of MET404 in GSC456/GSC23 cells transfected with METTL3 siRNAs.

g. Protein levels of MET404 in GSC456/GSC23 cells transfected with empty vector (EV), wild-type (WT) or mutated ALKBH5 (H204A).

h. GSCs transfected with empty vector (EV), wild-type YTHDF2 (WT) and mutated YTHDF2 (W432A and W486A) were subjected to RNA immunoprecipitation using anti-YTHDF2 antibody or IgG control and subsequent RT-qPCR analysis with circMET-specific primers. n=3 independent experiments. GSC456, EV vs WT, *P*=0.0060; WT vs W432A, *P*=0.011; WT vs W486A, *P*=0.016. GSC23, EV vs WT, *P*=3.79e-05; WT vs W432A, *P*=7.32e-04; WT vs W486A, *P*=2.74e-04.

i. Semiquantitative analysis of MET and MET404 expression levels in peritumour or tumour tissue based on immunoblot greyscale analysis in a cohort of 45 GBM patients.

j. Immunoblotting of MET and MET404 in GSC456 and GSC23 cells with stable MET404 KD using a MET N-terminal antibody. The specifically weakened band indicates the position of MET404.

k. Immunoblotting of MET and MET404 in GSCs using a MET N-terminal antibody.

l. Protein levels of MET404 in NHA, NSC, GBM cell lines (U251, U118 and SNB19) and GSC cell lines.

m. Illustration of CD133$^+$ and CD133$^-$ cell sorting from GBM tumour samples.

n. Relative circMET RNA levels in CD133$^+$ and CD133$^-$ cells sorted from independent GBM

samples (n=30). Two-sided paired *t* test, *P*=0.0042.

o. Representative immunoblotting of MET404 in $CD133^+$ and $CD133^-$ cells from 7 randomly chosen independent GBM samples.

p. Semiquantitative analysis of MET404 expression in $CD133^+$ and $CD133^-$ cells based on a greyscale analysis of the abovementioned GBM cohort of 30 independent samples. Two-sided paired *t* test, *P*=2.7546e-10.

The data in **d-g** and **j-l** were representative of three independent experiments. The data are presented as the mean±SD. Unpaired two-tailed Student's *t* test was used to determine the significance of differences between the indicated groups where applicable. **P*<0.05; ***P*<0.01; ****P*<0.001. Source data are provided as a Source Data file.

Supplementary Figure 5

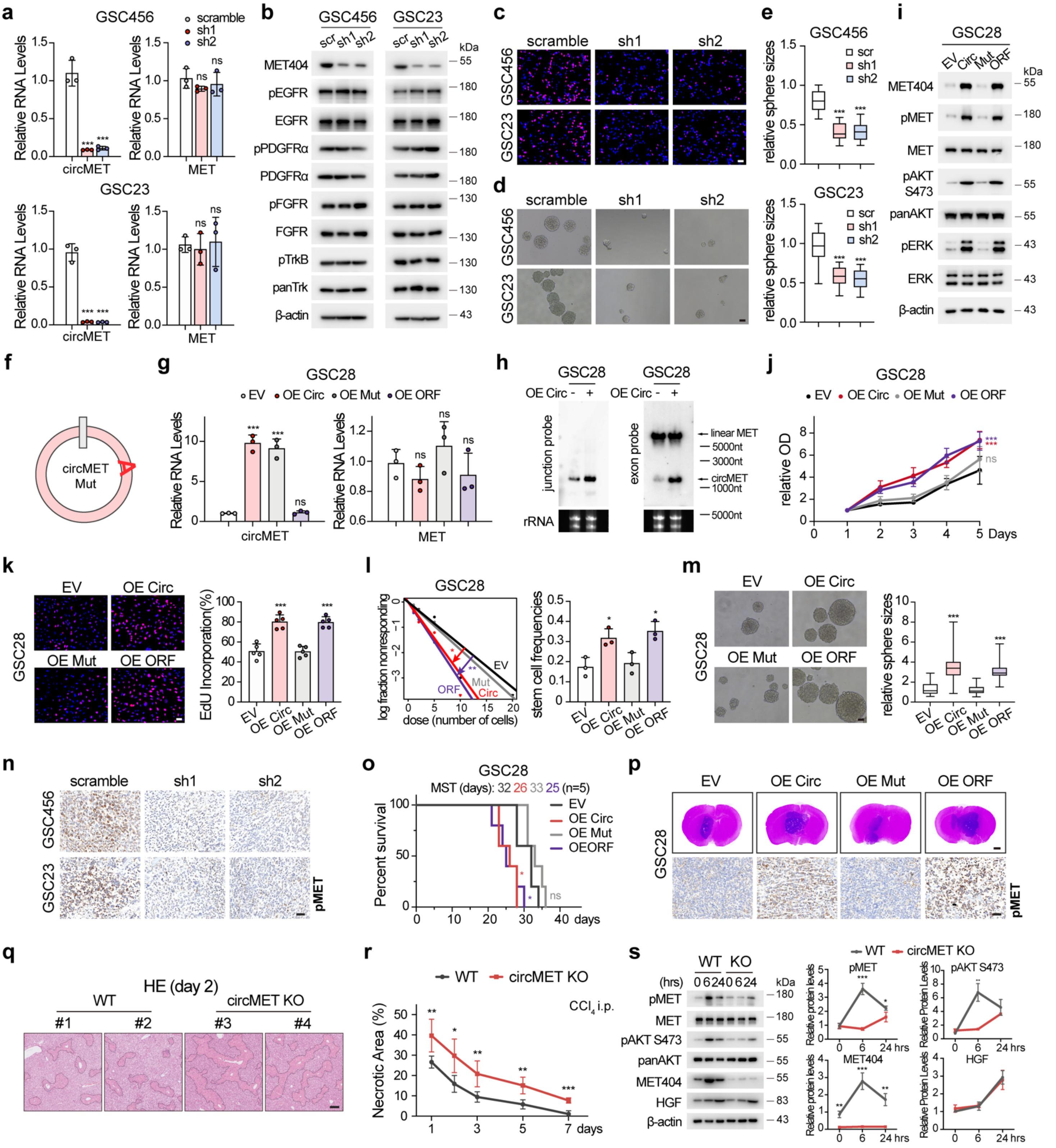

Supplementary Figure 5. MET404 boosts GBM tumorigenesis *in vitro* and *in vivo; The acute liver injury model indicates the critical role of MET404 in MET signalling* (related to Fig. 3)

a. Relative RNA levels of circMET and MET in GSC456/GSC23 cells with or without stable circMET KD. n=3 independent experiments. GSC456 circMET, scr vs sh1, *P*=5.03e-04; scr vs sh2, *P*=5.66e-04; GSC23 circMET, scr vs sh1, *P*=1.67e-04; scr vs sh2, *P*=1.64e-04.

b. Protein levels of pEGFR, EGFR, pPDGFRα, PDGFRα, pFGFR, FGFR, pTrkB, and panTrk in GSC456/GSC23 cells with or without stable circMET KD. Representative of three independent experiments.

c. Representative images of the EdU incorporation assay of control and stable circMET KD GSC456 and GSC23 cells. Representative of five independent experiments. Scale bar, 50 μm.

d. Live cell images of GSC456/GSC23 cells with or without stable circMET KD. Representative of three independent experiments. Scale bar, 50 μm.

e. Quantification of live cell images of GSC456/GSC23 cells with or without stable circMET KD. n=3 independent experiments. The data are presented as box plots containing the median (centre line), the first and third quartiles (box limits). The whiskers indicate the maxima and minima. GSC456, scr vs sh1, *P*=3.48e-28; scr vs sh2, *P*=5.88e-28; GSC23, scr vs sh1, *P*=1.25e-22; scr vs sh2, *P*=1.18e-24.

f. Illustration of the mutated circMET construct with insertion of adenine bases.

g. Relative RNA levels of circMET and MET in GSC28 cells with stable OE of empty vector (EV), circMET (Circ), mutated circMET (Mut), or MET404 ORF (ORF). n=3 independent experiments. EV vs OE Circ, *P*=1.13e-04; EV vs OE Mut, *P*=2.74e-04.

h. Northern blotting using junction probes (left) or exon probes (right) was performed to validate the OE of circMET RNA in GSC28 cells. Representative of three independent experiments.

i. Protein levels of phospho-MET and downstream phospho-AKT (S473) and phospho-ERK in GSC28 cells with the indicated modifications. Representative of three independent experiments.

j. Cell proliferation assay of GSC28 cells with the indicated modifications. n=5 independent experiments. Two-way ANOVA. EV vs OE Circ, *P*=3.29e-10; EV vs OE ORF, *P*=3.02e-10.

k. Representative images (left) and quantification (right) of the EdU incorporation assay of GSC28 cells with the indicated modifications. n=5 independent experiments. Scale bar, 50 μm. EV vs OE Circ, *P*=1.95e-04; EV vs OE ORF, *P*=1.06e-04.

l. Left, limited dilution assay (LDA) analysis of GSC28 cells with the indicated modifications. Right, stem cell frequencies obtained from the LDA analysis of GSC28 cells with the indicated modifications. n=3 independent experiments. EV vs OE Circ, *P*=0.020; EV vs OE ORF, *P*=0.010.

m. Live cell images (left) and corresponding quantification (right) of GSC28 cells with the indicated modifications. n=3 independent experiments. Scale bar, 50 μm. The quantification data are presented as box plots containing the median (centre line), the first and third quartiles (box limits). The whiskers indicate the maxima and minima. EV vs OE Circ, *P*=9.84e-19; EV vs OE ORF, *P*=9.95e-21.

n. Representative phospho-MET IHC images of brain slices from mice implanted with GSC456/GSC23 cells with or without stable circMET KD (n=5 per group). Scale bar, 50 μm.

o. Survival analysis of mice implanted with GSC28 cells with the indicated modifications (n=5 per group). Log-rank test. EV vs OE Circ, *P*=0.017; EV vs OE ORF, *P*=0.025.

p. Top, representative H&E-stained brain slices of mice implanted with GSC28 cells with the indicated modifications (n=5 per group). Scale bar, 1 mm. Bottom, representative phospho-MET IHC images from the same cohort of mice. Scale bar, 50 μm.

q. Representative H&E-stained images of the CCl_4-induced acute liver injury model using wild-type or circMET KO mice on day 2 (n=5 per group). The dashed lines indicate the border of the necrotic area. Scale bar, 200 μm.

r. Quantification of the liver necrotic area of the CCl_4-induced acute liver injury model using wild-type or circMET KO mice (n=5 per group at each time point). Day 1, *P*=0.0094; Day 2, *P*=0.010; Day 3, *P*=0.0060; Day 5, *P*=0.0021; Day 7, *P*=7.85e-05.

s. Left, immunoblot of phospho-MET and downstream signals in liver tissue of wild-type or circMET KO mice collected at the indicated time points after intraperitoneal injection of CCl_4. Representative of three independent experiments. Right, quantitative analysis of phospho-MET, MET404, phospho-AKT(S473) and HGF protein levels in the left panel by greyscale analysis (n=3 per group at each time point). pMET, 6h *P*=2.92e-04; 24h *P*=0.044. pAKT S473, 6h *P*=0.0020; MET404, 0h *P*=0.0028; 6h *P*=7.12e-04; 24h *P*=0.0016.

The data are presented as the mean±SD. Unpaired two-tailed Student's *t* test was used to determine the significance of the differences between the indicated groups where applicable. **P*<0.05; ***P*<0.01; ****P*<0.001. Source data are provided as a Source Data file.

Supplementary Figure 6

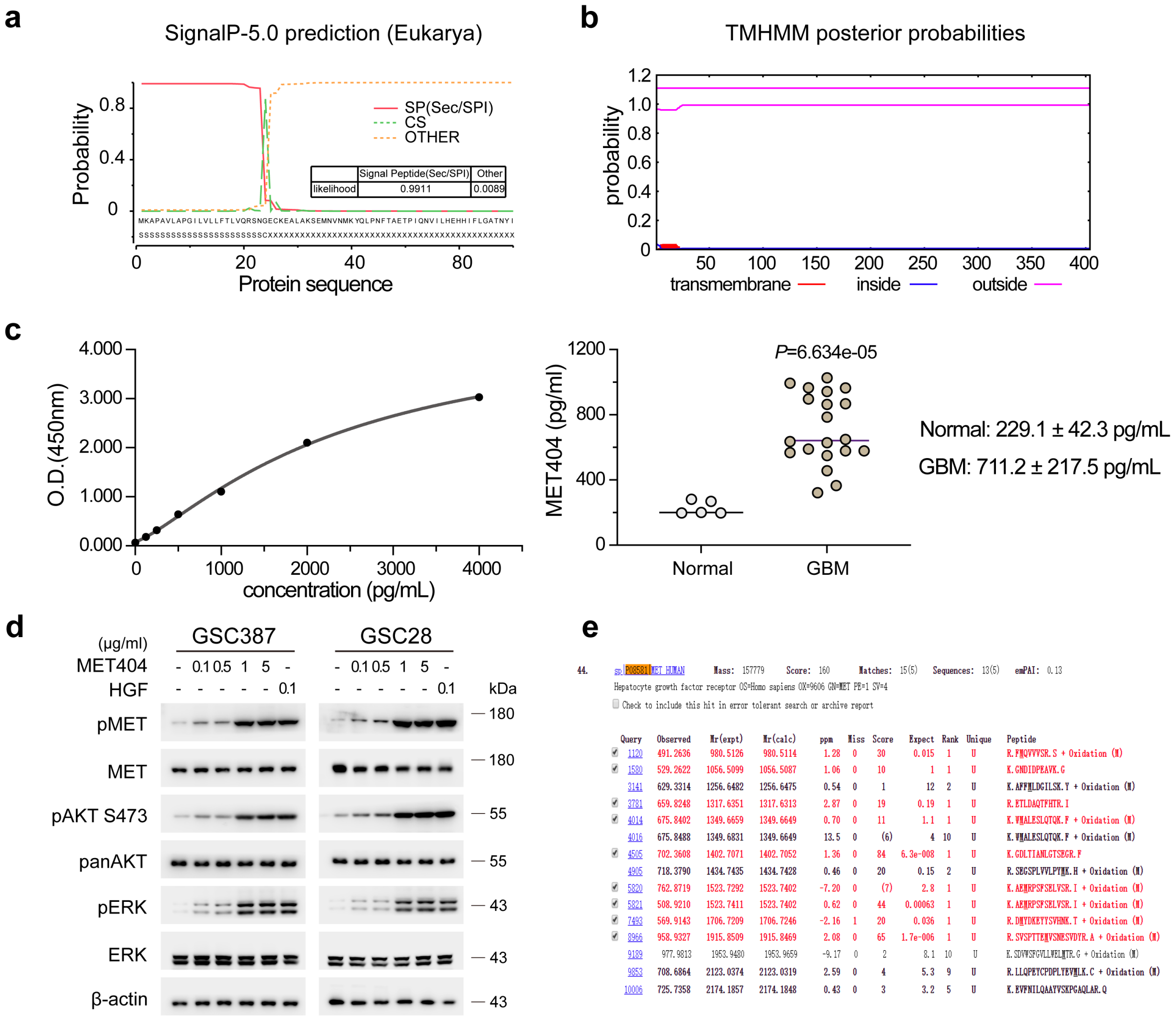

Supplementary 6. Secreted MET404 activates MET signalling by interacting with MET (related to Fig. 5)

a. MET404 secretion potential predicted by SignalIP-5.0 [https://services.healthtech.dtu.dk/service.php?SignalP-5.0].

b. MET404 localization probabilities predicted by TMHMM [https://services.healthtech.dtu.dk/service.php?TMHMM-2.0].

c. Left, standard curve for quantification of the MET404 levels in CSF samples. Right, scatter plot of the MET404 levels in independent GBM (n=20) and control (n=5) CSF samples. Unpaired two-tailed Student's *t* test, *P*=6.634e-05.

d. Protein levels of phospho-MET and downstream phospho-AKT (S473) and phospho-ERK in GSC387 and GSC28 cells treated with gradient concentrations of MET404. HGF was used as a

positive control. Representative of three independent experiments.

e. Mascot search results of the IP/MS analysis revealed that MET sequences were pulled down by an anti-MET404 antibody.

Source data are provided as a Source Data file.

Supplementary Figure 7

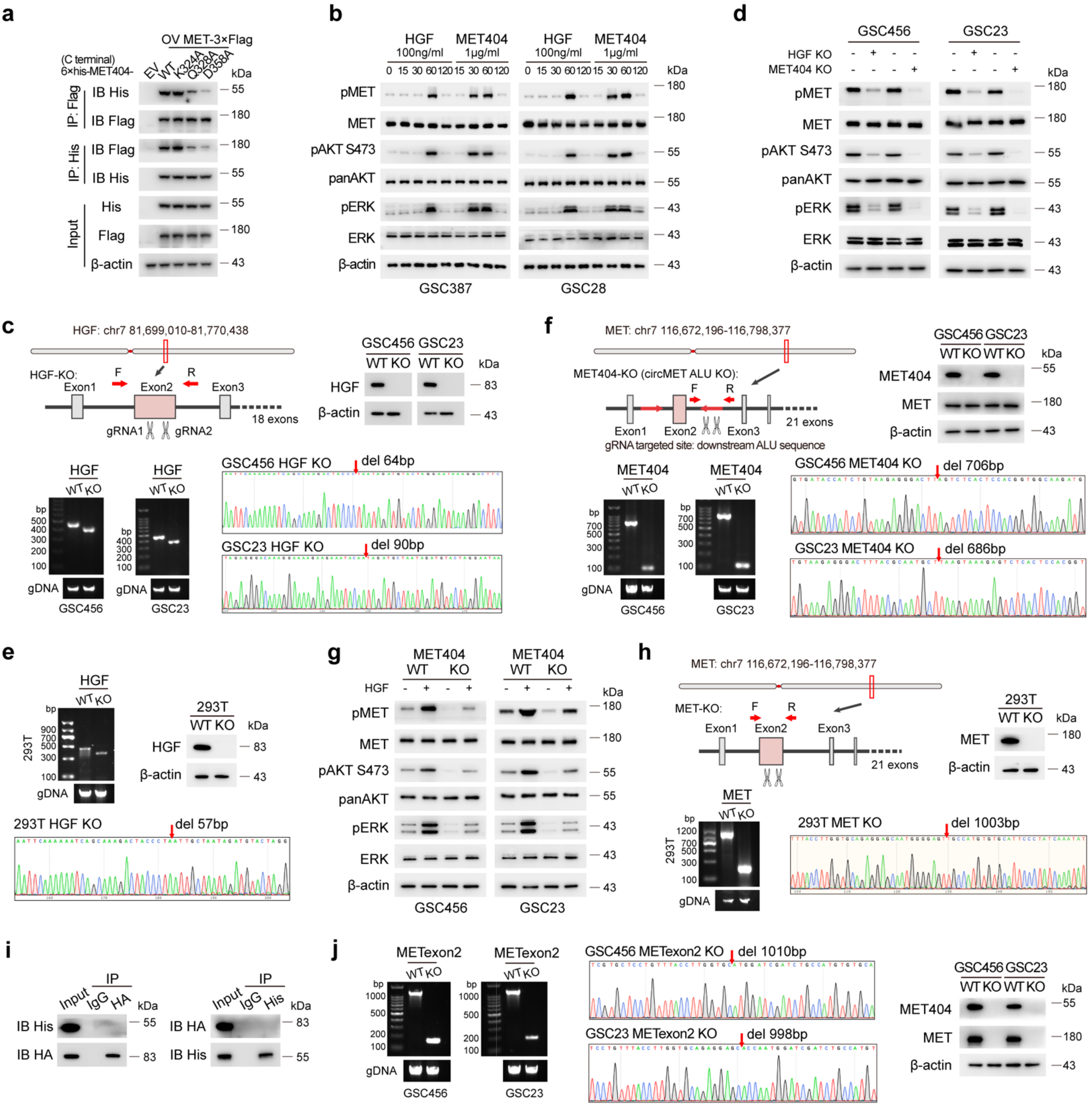

Supplementary Figure 7. Key points on MET404 mediating the interaction with MET; verification of KO cell lines; phospho-MET levels in KO cell lines (related to Fig. 6)

a. 293T cells were transfected with wild-type MET404, point-mutated MET404 (with a His tag at the C-terminus) or empty vector, along with flag-tagged MET. Whole-cell lysates were subjected to immunoprecipitation with anti-His or anti-Flag antibodies, followed by immunoblotting with anti-His or anti-Flag antibodies.

b. Protein levels of phospho-MET and downstream phospho-AKT (S473) and phospho-ERK in GSC387 and GSC28 cells detected at the indicated timepoints after stimulation with HGF or MET404.

c. Upper left, CRISPR/Cas9-mediated HGF KO strategy and primer design for PCR validation. Upper right, immunoblot for HGF in wild-type (WT) or HGF KO GSC456/GSC23 cells. Lower left, DNA gel electrophoresis of PCR for HGF in WT or HGF KO GSC456/GSC23 cells. Lower right, Sanger sequencing of PCR products for HGF in the indicated cells.

d. Protein levels of phospho-MET and downstream phospho-AKT (S473) and phospho-ERK in WT, HGF KO or MET404 KO GSC456/GSC23 cells.

e. Upper left, DNA gel electrophoresis of PCR for HGF in WT or HGF KO 293T cells. Upper right, immunoblot for HGF in WT or HGF KO 293T cells. Bottom, Sanger sequencing of PCR products for HGF in the indicated cells.

f. Upper left, CRISPR/Cas9-mediated MET404 KO (circMET ALU KO) strategy and primer design for PCR validation. Upper right, immunoblot of MET404 in WT or MET404 KO GSC456/GSC23 cells. Lower left, DNA gel electrophoresis of PCR for the circMET downstream *Alu* sequence in WT or MET404 KO GSC456/GSC23 cells. Lower right, Sanger sequencing of PCR products for circMET downstream of the *Alu* sequence in the indicated cells.

g. Protein levels of phospho-MET and downstream phospho-AKT (S473) and phospho-ERK in GSC456/GSC23 wild-type (WT) or MET404 KO cells with or without 1 hour of HGF stimulation (100 ng/ml).

h. Upper left, CRISPR/Cas9-mediated MET exon 2 KO strategy and primer design for PCR validation. Upper right, immunoblot of MET in WT or MET KO 293T cells. Lower left, DNA gel electrophoresis of PCR for MET exon 2 in the indicated cells. Lower right, Sanger sequencing of PCR products for MET exon2 in the indicated cells.

i. HGF-HA and MET404-6×His were transfected into 293T MET KO cells. Whole-cell lysates were subjected to immunoprecipitation with anti-His or anti-HA antibodies, followed by immunoblotting with anti-His or anti-HA antibodies.

j. Left, DNA gel electrophoresis of PCR for MET exon 2 in WT or MET exon 2 KO GSC456/GSC23 cells. Middle, Sanger sequencing of PCR products for *MET* exon2 in the indicated cells. Right, immunoblot of MET404 and MET in the indicated cells.

The data are representative of three independent experiments. Source data are provided as a Source Data file.

Supplementary Figure 8

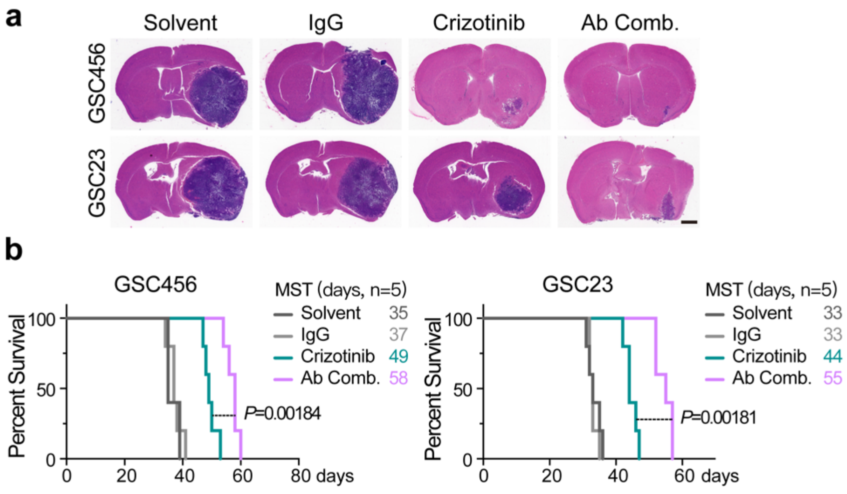

Supplementary Figure 8. Comparison of antibody combination therapy with crizotinib against GSC xenografts.

a. Representative H&E-stained brain slices from mice implanted with GSC456 or GSC23 and treated with antibody combination therapy (anti-MET404 and onartuzumab) or crizotinib (n=5 per group). Scale bar, 1 mm.

b. Survival analysis of mice treated with the indicated therapeutic strategy (n=5 per group). Log-rank test.

Source data are provided as a Source Data file.

Supplementary Figure 9

a

Genetic alterations of a panel of glioblastoma stem cells (GSC)

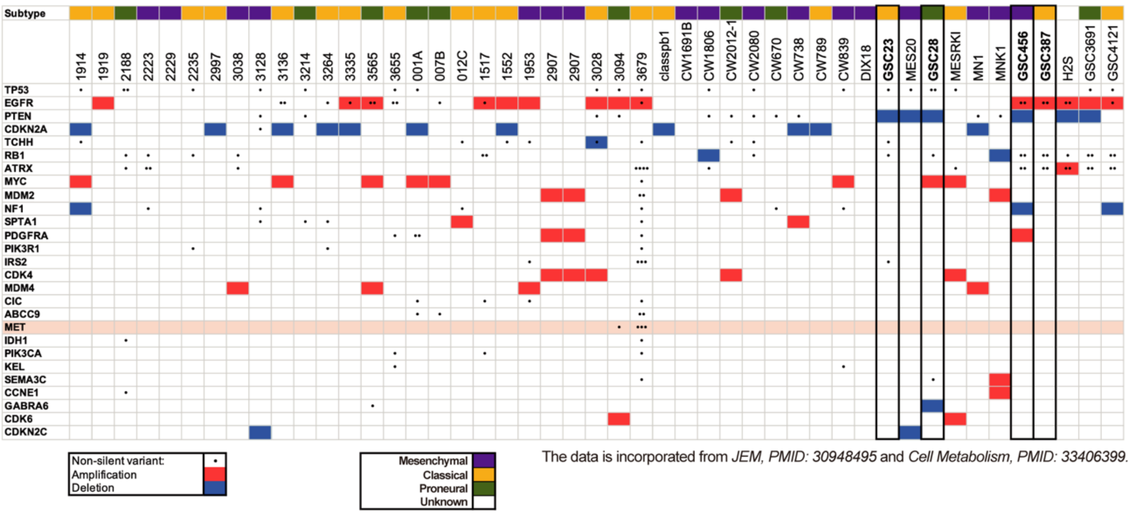

b

Transcriptional Subtype of GSCs used in the manuscript

GSC	Patient Age, Gender	Tumor Grade	Transcriptional Subtype
GSC387	76, female	WHO IV	Classical
GSC456	8, female	WHO IV	Mesenchymal
GSC28	Unknown	WHO IV	Proneural
GSC23	63, male	WHO IV	Classical

c

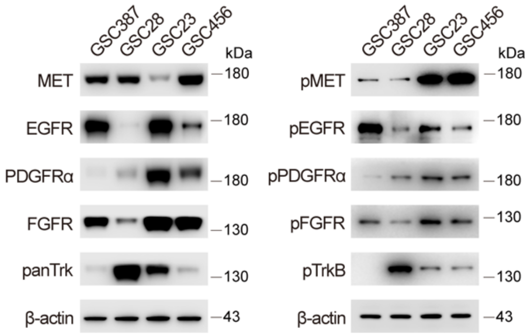

Supplementary Figure 9. Supplementary Data of glioblastoma stem cells (GSC).

a. Genetic alterations of a panel of GSCs including GSCs used in this manuscript (highlighted in black boxes) and two published reports[1,2]. Amplification, deletion and non-silent variant are shown in the diagram. Transcriptional subtypes of the GSCs are also shown in different colours: Mesenchymal (purple), Classical (Yellow), Proneural (Green), Unknown (White).

b. Table listing transcriptional subtypes and patient information of the used GSCs in the manuscript.

c. The protein levels of MET, EGFR, PDGFRα, FGFR, panTrk and their phosphorylated forms in the indicated GSCs. Representative of three independent experiments.

Source data are provided as a Source Data file.

Supplementary Table 1 Contact list of docking analysis

Protein	Residue	Protein	Residue	Type	Energy (Kcal/mol)	Distance (Å)
MET	Trp586.NE1	MET404	Tyr41.O	Hydrogen bond	-1	2.979
MET	Asp543.O	MET404	Gln42.NE2	Hydrogen bond	-2.7	2.905
MET	His542.O	MET404	Asn45.ND2	Hydrogen bond	-5.2	2.978
MET	His542.ND1	MET404	Thr47.OG1	Hydrogen bond	-3.1	2.85
MET	Ser650.OG	MET404	Glu75.OE1	Hydrogen bond	-2.4	2.841
MET	Arg602.NH2	MET404	Ala320.O	Hydrogen bond	-11.4	2.845
MET	Val603.O	MET404	Tyr321.OH	Hydrogen bond	-1.1	2.896
MET	Ser609.OG	MET404	Val322.O	Hydrogen bond	-1.9	2.714
MET	Glu608.OE1/OE2	MET404	Lys324.NZ	Salt bridge	-41.955	2.745
MET	Thr611.OG1	MET404	Lys324.O	Hydrogen bond	-2.6	2.896
MET	Gly578.O	MET404	Ala327.N	Hydrogen bond	-2.6	3.016
MET	Arg580.NH1/NH2	MET404	Gln328.OE1	Hydrogen bond	-10.2	2.673
MET	Glu576.OE2	MET404	Asn338.N	Hydrogen bond	-7.4	3.396
MET	Lys595.NZ	MET404	Asp358.OD2	Salt bridge	-22.616	2.728

Supplementary Table 2 Gene Fragments for CircMET Overexpression Plasmid Construction

circMET overexpression
**AGTGCTGAGATTACAGGCGTGAGCCACCACCCCCGGCCCACTTTTTGTAAAGGTACGTACTA**<br>**ATGACTTTTTTTTTATACTTCAG**ATAAACCTCTCATAATGAAGGCCCCCGCTGTGCTTGCACCTGG<br>CATCCTCGTGCTCCTGTTTACCTTGGTGCAGAGGAGCAATGGGGAGTGTAAAGAGGCACTAGCAAA<br>GTCCGAGATGAATGTGAATATGAAGTATCAGCTTCCCAACTTCACCGCGGAAACACCCATCCAGAA<br>TGTCATTCTACATGAGCATCACATTTTCCTTGGTGCCACTAACTACATTTATGTTTTAAATGAGGAA<br>GACCTTCAGAAGGTTGCTGAGTACAAGACTGGGCCTGTGCTGGAACACCCAGATTGTTTCCCATGT<br>CAGGACTGCAGCAGCAAAGCCAATTTATCAGGAGGTGTTTGGAAAGATAACATCAACATGGCTCT<br>AGTTGTCGACACCTACTATGATGATCAACTCATTAGCTGTGGCAGCGTCAACAGAGGGACCTGCCA<br>GCGACATGTCTTTCCCCACAATCATACTGCTGACATACAGTCGGAGGTTCACTGCATATTCTCCCCA<br>CAGATAGAAGAGCCCAGCCAGTGTCCTGACTGTGTGGTGAGCGCCCTGGGAGCCAAAGTCCTTTCA<br>TCTGTAAAGGACCGGTTCATCAACTTCTTTGTAGGCAATACCATAAATTCTTCTTATTTCCCAGATC<br>ATCCATTGCATTCGATATCAGTGAGAAGGCTAAAGGAAACGAAAGATGGTTTTATGTTTTTGACGG<br>ACCAGTCCTACATTGATGTTTTACCTGAGTTCAGAGATTCTTACCCCATTAAGTATGTCCATGCCTT<br>TGAAAGCAACAATTTTATTTACTTCTTGACGGTCCAAAGGGAAACTCTAGATGCTCAGACTTTTCAC<br>ACAAGAATAATCAGGTTCTGTTCCATAAACTCTGGATTGCATTCCTACATGGAAATGCCTCTGGAG<br>TGTATTCTCACAGAAAAGAGAAAAAAGAGATCCACAAAGAAGGAAGTGTTTAATATACTTCAGGC<br>TGCGTATGTCAGCAAGCCTGGGGCCCAGCTTGCTAGACAAATAGGAGCCAGCCTGAATGATGACAT<br>TCTTTTCGGGGTGTTCGCACAAAGCAAGCCAGATTCTGCCGAACCAATGGATCGATCTGCCATGTG<br>TGCATTCCCTATCAAATATGTCAACGACTTCTTCAACAAGATCGTCAACAAAAACAATGTGAGATG<br>TCTCCAGCATTTTTACGGACCCAATCATGAGCACTGCTTTAATAGG**GTAAGAAGCAAGGAAAAGA**<br>**ATTAGGCTCGGCACGGTAGCTCACACCTGTAATCCCAGCA**

**The underlined part denotes the cyclization sequences.*

circMET Mut

AGTGCTGAGATTACAGGCGTGAGCCACCACCCCCGGCCCACTTTTTGTAAAGGTACGTACTA
ATGACTTTTTTTTTATACTTCAGATAAACCTCTCATAATGAAGGCCCCCGCTGTGCTTGCACCTGG
CATCCTCGTGCTCCTGTTTACCTTGGTGCAGAGGAGCAATGGGGAGTGTAAAGAGGCACTAGCAAA
GTCCGAGATGAATGTGAATATGAAAGTATCAGCTTCCCAACTTCACCGCGGAAACACCCATCCAGA
ATGTCATTCTACATGAGCATCACATTTTCCTTGGTGCCACTAACTACATTTATGTTTTAAATGAGGA
AGACCTTCAGAAGGTTGCTGAGTACAAGACTGGGCCTGTGCTGGAACACCCAGATTGTTTCCCATG
TCAGGACTGCAGCAGCAAAGCCAATTTATCAGGAGGTGTTTGGAAAGATAACATCAACATGAGCT
CTAGTTGTCGACACCTACTATGATGATCAACTCATTAGCTGTGGCAGCGTCAACAGAGGGACCTGC
CAGCGACATGTCTTTCCCCACAATCATACTGCTGACATACAGTCGGAGGTTCACTGCATATTCTCCC
CACAGATAGAAGAGCCCAGCCAGTGTCCTGACTGTGTGGTGAGCGCCCTGGGAGCCAAAGTCCTTT
CATCTGTAAAGGACCGGTTCATCAACTTCTTTGTAGGCAATACCATAAATTCTTCTTATTTCCCAGA
TCATCCATTGCATTCGATATCAGTGAGAAGGCTAAAGGAAACGAAAGATGGTTTTATGATTTTTGA
CGGACCAGTCCTACATTGATGTTTTACCTGAGTTCAGAGATTCTTACCCCATTAAGTATGTCCATGC
CTTTGAAAGCAACAATTTTATTTACTTCTTGACGGTCCAAAGGGAAACTCTAGATGCTCAGACTTTT
CACACAAGAATAATCAGGTTCTGTTCCATAAACTCTGGATTGCATTCCTACATGGAAATGACCTCT
GGAGTGTATTCTCACAGAAAAGAGAAAAAAGAGATCCACAAAGAAGGAAGTGTTTAATATACTTC
AGGCTGCGTATGTCAGCAAGCCTGGGGCCCAGCTTGCTAGACAAATAGGAGCCAGCCTGAATGAT
GACATTCTTTTCGGGGTGTTCGCACAAAGCAAGCCAGATTCTGCCGAACCAATGGATCGATCTGCC
ATGTGTGCATTCCCTATCAAATATGTCAACGACTTCTTCAACAAGATCGTCAACAAAAACAATGTG
AGATGTCTCCAGCATTTTTACGGACCCAATCATGAGCACTGCTTTAATAGG**GTAAGAAGCAAGGA**
AAAGAATTAGGCTCGGCACGGTAGCTCACACCTGTAATCCCAGCA

**The underlined part denotes the cyclization sequences. The inserted adenine bases (A) are highlighted in red.*

circMET ORF

ATGAAGGCCCCCGCTGTGCTTGCACCTGGCATCCTCGTGCTCCTGTTTACCTTGGTGCAGAGGAGC
AATGGGGAGTGTAAAGAGGCACTAGCAAAGTCCGAGATGAATGTGAATATGAAGTATCAGCTTCC
CAACTTCACCGCGGAAACACCCATCCAGAATGTCATTCTACATGAGCATCACATTTTCCTTGGTGCC
ACTAACTACATTTATGTTTTAAATGAGGAAGACCTTCAGAAGGTTGCTGAGTACAAGACTGGGCCT
GTGCTGGAACACCCAGATTGTTTCCCATGTCAGGACTGCAGCAGCAAAGCCAATTTATCAGGAGGT
GTTTGGAAAGATAACATCAACATGGCTCTAGTTGTCGACACCTACTATGATGATCAACTCATTAGC
TGTGGCAGCGTCAACAGAGGGACCTGCCAGCGACATGTCTTTCCCCACAATCATACTGCTGACATA
CAGTCGGAGGTTCACTGCATATTCTCCCCACAGATAGAAGAGCCCAGCCAGTGTCCTGACTGTGTG
GTGAGCGCCCTGGGAGCCAAAGTCCTTTCATCTGTAAAGGACCGGTTCATCAACTTCTTTGTAGGC
AATACCATAAATTCTTCTTATTTCCCAGATCATCCATTGCATTCGATATCAGTGAGAAGGCTAAAGG
AAACGAAAGATGGTTTTATGTTTTTGACGGACCAGTCCTACATTGATGTTTTACCTGAGTTCAGAGA
TTCTTACCCCATTAAGTATGTCCATGCCTTTGAAAGCAACAATTTTATTTACTTCTTGACGGTCCAA
AGGGAAACTCTAGATGCTCAGACTTTTCACACAAGAATAATCAGGTTCTGTTCCATAAACTCTGGA
TTGCATTCCTACATGGAAATGCCTCTGGAGTGTATTCTCACAGAAAAGAGAAAAAAGAGATCCACA
AAGAAGGAAGTGTTTAATATACTTCAGGCTGCGTATGTCAGCAAGCCTGGGGCCCAGCTTGCTAGA
CAAATAGGAGCCAGCCTGAATGATGACATTCTTTTCGGGGTGTTCGCACAAAGCAAGCCAGATTCT
GCCGAACCAATGGATCGATCTGCCATGTGTGCATTCCCTATCAAATATGTCAACGACTTCTTCAACA

AGATCGTCAACAAAAACAATGTGAGATGTCTCCAGCATTTTTACGGACCCAATCATGAGCACTGCT TTAATAGGATAAACCTCTCA**<u>TAATGA</u>**

**The underlined part denotes the start codon (ATG) and the stop codon (TAA, TGA).*

Supplementary Table 3 RNA oligos

siRNA	sense 5'-3'	antisense 5'-3'
circMET-siRNA-NC	GCUUUAAAUUCAUUAUCGUGAtt	UCACGAUAAUGAAUUUAAAGCtt
circMET-siRNA1	GCUUUAAUAGGAUAAACCUCUtt	AGAGGUUUAUCCUAUUAAAGCtt
circMET-siRNA2	UAAUAGGAUAAACCUCUCAUAtt	UAUGAGAGGUUUAUCCUAUUAtt
YTHDF1-siRNA1	CGUUACAUCAGAAGGAUACAGUUCAtt	UGAACUGUAUCCUUCUGAUGUAACGtt
YTHDF1-siRNA2	GGACAAAUGUGAACAUGCCAGUUUCtt	GAAACUGGCAUGUUCACAUUUGUCCtt
YTHDF2-siRNA1	UACUGAUUAAGUCAGGAUUAAtt	UUAAUCCUGACUUAAUCAGUAtt
YTHDF2-siRNA2	UACUGAUUAAGUCAGGAUUAAtt	GUUAUAGUUAUUAAUGGACCGtt
YTHDF3-siRNA1	GAUAAGUGGAAGGGCAAAUUUtt	AAAUUUGCCCUUCCACUUAUCtt
YTHDF3-siRNA2	UAAGUCAAAGAAGACGUAUUAtt	UAAUACGUCUUCUUUGACUUAtt
METTL3-siRNA1	GCUACCUGGACGUCAGUAUtt	AUACUGACGUCCAGGUAGCtt
METTL3-siRNA2	GGUUGGUGUCAAAGGAAAUtt	AUUUCCUUUGACACCAACCtt
HGF-siRNA1	CGACAAGGGCUUUGAUGAUAAUUAU	AUAAUUAUCAUCAAAGCCCUUGUCG
HGF-siRNA2	CAUAUCUGCGGAGGAUCAUUGAUAA	UUAUCAAUGAUCCUCCGCAGAUAUG
EGFR-siRNA1	CGCAAAGUGUGUAACGGAAUAGGUA	UACCUAUUCCGUUACACACUUUGCG
EGFR-siRNA2	CACCGUGGCUUGCAUUGAUAGAAAU	AUUUCUAUCAAUGCAAGCCACGGUG
Ctrl-siRNA	UUCUCCGAACGUGUCACGUUtt	AACGUGACACGUUCGGAGAAtt

sgRNA	gRNA1	gRNA2	gRNA3	gRNA4
HGF KO	TAGTACATCTATT AGCACAT	TGCTGGATCTATTT TGATTA	/	/
MET exon2 KO	GAGCAATGGGGAG TGTAAAG	GGCAGATCGATCC ATTGGTT	/	/
circMET ALU KO	ACGCAATGCTTTA GTGGGCG	TTCAACTTGAAAT CATGGAT	CCACTGGGATTAC GATCCTG	TAAAGAGTCTCAC TCCACGG

**circMET FISH probe**	5' CY3 - TCATTATGAGAGGTTTATCCTATTAAAGCAGTGCTC -3'
**circMET Northern-junction probe**	5'-GGGGGCCTTCATTATGAGAGGTTTATCCTATTAAAGCAGTGCTCATGATT-3'
**circMET Northern-exon-probe**	5'-AGATGAAAGGACTTTGGCTCCCAGGGCGCTCACCACACAGTCAGGACACT-3'

Supplementary Table 4 PCR Primers

Primers	Forward	Reverse
circMET (qPCR)	GATTCTGCCGAACCAATGGA	CACTCCCCATTGCTCCTCTG
MET (qPCR)	CCCAATCATGAGCACTGCTTTA	GAGGACTTCGCTGAATTGAC
circSMO (qPCR)	TCCTCACTGTGGCAATCCTT	TGATGTTCTGCACCTCATTCTC
circHIPK3 (qPCR)	TCGGCCAGTCATGTATCAAA	ACCAAGACTTGTGAGGCCAT
MYC (qPCR)	GGCTCCTGGCAAAAGGTCA	CTGCGTAGTTGTGCTGATGT
METTL3 (qPCR)	TTGTCTCCAACCTTCCGTAGT	CCAGATCAGAGAGGTGGTGTAG
YTHDF2 (qPCR)	GTCAGGGACAAAAGCCTCCG	GACCTTTTGGTCTCTGCTCCA
HGF (PCR)	CTCACTCTGCTCCTAACCCC	ACTGAGAGCTAAGTCTTCGTTTGT
MET (PCR)	CTCATAATGAAGGCCCCCGC	GTGCTCATGATTGGGTCCGTA
circMET (PCR)	GCCTTTCAACAGCACTCACC	CTTATCCCTCCTTTCCATCTTGC

Supplementary References:

1. Mack, S. C. *et al.* Chromatin landscapes reveal developmentally encoded transcriptional states that define human glioblastoma. *J Exp Med* **216**, 1071–1090 (2019).
2. Huang, N. *et al.* An Upstream Open Reading Frame in Phosphatase and Tensin Homolog Encodes a Circuit Breaker of Lactate Metabolism. *Cell Metabolism* **33**, 128-144.e9 (2021).
