# Supplementary material for: Circular RNA encoded MET variant promotes glioblastoma tumorigenesis
Source: Nat Commun. 2023 Jul 25;14:4467. doi: 10.1038/s41467-023-40212-1 (PMC10368723; doi:10.1038/s41467-023-40212-1)
Supplement: Supplementary file 3 — Description of Additional Supplementary Files [file 41467_2023_40212_MOESM3_ESM.pdf]

Description of Additional Supplementary Files

Supplementary Data 1: circRNA expression of m^6A-seq. Table listing identified circRNAs and their expression in the m^6A-seq of 10 human GBM samples.

Supplementary Data 2: Pathway analysis of source genes of m6A-seq identified circRNAs. Table listing enriched pathways generated from KEGG analysis of source genes of m6A-modified circRNAs. Hypergeometric test. The exact *P* values are provided in the dataset.

Supplementary Data 3: Differentially expressed circRNAs of RNC-seq. Table listing the differentially expressed circRNAs between five patient-derived glioblastoma stem cells (GSCs) and two control neural stem cells (NSCs). A total of 1,605 differentially expressed RNC-circRNAs ($log_2FC>2$, $P<0.05$), with 349 upregulated and 1,256 downregulated in GSCs.

Supplementary Data 4: circRNA expression of YTHDF2 RIP-seq. Table listing identified 255 circRNAs and their expression in the YTHDF2 RIP-seq of two GSCs.

Supplementary Data 5: Pathway analysis of RNA-seq in circMET stably overexpressed (OE) or knocked down (KD) cells. Table listing enriched pathways based on KEGG analysis of differentially expressed genes identified in bulk RNA-seq comparing circMET KD GSC23 and the control cells, or circMET OE GSC28 and the control cells. Hypergeometric test. The exact *P* values are provided in the dataset.

Supplementary Data 6: Candidate binding partners of MET404 identified by IP-MS. Table listing a total of 196 candidate proteins as potential binding partners of mET404 as revealed by mass spectrometry analysis of MET404 immunoprecipitated proteins.

Supplementary Data 7: Pathway analysis of MET404 binding partners. Table listing enriched pathways based on Reactome pathway analysis of the candidate binding partners of MET404. Hypergeometric test. The exact *P* values are provided in the dataset.
