# Supplementary material for: Circular RNA encoded MET variant promotes glioblastoma tumorigenesis
Source: Nat Commun. 2023 Jul 25;14:4467. doi: 10.1038/s41467-023-40212-1 (PMC10368723; doi:10.1038/s41467-023-40212-1)
Supplement: Supplementary file 11 — Reporting Summary [file 41467_2023_40212_MOESM11_ESM.pdf]

nature portfolio

Corresponding author(s): Xiuxing Wang, Fan Bai, Yu Shi, Nu Zhang

Last updated by author(s): Jun 29, 2023

Reporting Summary

Nature Portfolio wishes to improve the reproducibility of the work that we publish. This form provides structure for consistency and transparency in reporting. For further information on Nature Portfolio policies, see our Editorial Policies and the Editorial Policy Checklist.

Statistics

For all statistical analyses, confirm that the following items are present in the figure legend, table legend, main text, or Methods section.

n/a	Confirmed	
☐	☒	The exact sample size (*n*) for each experimental group/condition, given as a discrete number and unit of measurement
☐	☒	A statement on whether measurements were taken from distinct samples or whether the same sample was measured repeatedly
☐	☒	The statistical test(s) used AND whether they are one- or two-sided<br>*Only common tests should be described solely by name; describe more complex techniques in the Methods section.*
☐	☒	A description of all covariates tested
☐	☒	A description of any assumptions or corrections, such as tests of normality and adjustment for multiple comparisons
☐	☒	A full description of the statistical parameters including central tendency (e.g. means) or other basic estimates (e.g. regression coefficient) AND variation (e.g. standard deviation) or associated estimates of uncertainty (e.g. confidence intervals)
☐	☒	For null hypothesis testing, the test statistic (e.g. *F*, *t*, *r*) with confidence intervals, effect sizes, degrees of freedom and *P* value noted<br>*Give P values as exact values whenever suitable.*
☒	☐	For Bayesian analysis, information on the choice of priors and Markov chain Monte Carlo settings
☒	☐	For hierarchical and complex designs, identification of the appropriate level for tests and full reporting of outcomes
☐	☒	Estimates of effect sizes (e.g. Cohen's *d*, Pearson's *r*), indicating how they were calculated

Our web collection on statistics for biologists contains articles on many of the points above.

Software and code

Policy information about availability of computer code

Data collection	RSEM (version 1.2.19), ZEISS Zen (black edition version 2), QuantStudio 5 Operating Software (version 1.4.0), FACS Diva software (version 7)
Data analysis	fastp (version 0.20.0), Bowtie2 (version 2.2.8), HISAT2 (version 2.1.1), R language (version 3.2.19), GraphPad Prism (version 8.0), ImageJ (version 1.52), edge R package (version 3.12.1), SKanIt (version 6.1), Image Lab (version 6.0.1), Microsoft Excel (Version 16.74), Cytoscape (version 3.9.1), find_circ (version 1.2), CIRIquant (version 2.1.1)

For manuscripts utilizing custom algorithms or software that are central to the research but not yet described in published literature, software must be made available to editors and reviewers. We strongly encourage code deposition in a community repository (e.g. GitHub). See the Nature Portfolio guidelines for submitting code & software for further information.

Data

Policy information about availability of data

All manuscripts must include a data availability statement. This statement should provide the following information, where applicable:

- Accession codes, unique identifiers, or web links for publicly available datasets
- A description of any restrictions on data availability
- For clinical datasets or third party data, please ensure that the statement adheres to our policy

The m6A-seq data of human GBM samples have been deposited in the National Genomics Data Centre (GSA-human database) under the accession code HRA002365 [https://ngdc.cncb.ac.cn/gsa-human/browse/HRA002365]. The RNC-seq and YTHDF2 RIP-seq data have been deposited in the NCBI database under the

accession ID PRJNA973050 [https://www.ncbi.nlm.nih.gov/bioproject/973050]. The remaining data are available within the Article, Supplementary Information and Source data file. Source data are provided with this paper.

Human research participants

Policy information about studies involving human research participants and Sex and Gender in Research.

Reporting on sex and gender	Sex and gender were identified as insignificant factor in prognosis analysis. Source data disaggregated for sex has been collected and will be provided with the revised manuscript, if applicable. All pathologically diagnosed glioma samples and adjacent normal brain tissues used in this study were collected from the Department of Neurosurgery of the 1st Affiliated Hospital of Sun Yat-sen University with informed consent.
Population characteristics	71 Frozen human glioma samples (35 males and 36 females, aged from 19 to 77) were used. No personal information about the identity of the patients was made accessible to the researchers.
Recruitment	A series of 71 human glioma frozen samples were collected with patient consent from the biobank of the Department of Neurosurgery of the First Affiliated Hospital of Sun Yat-sen University. No self-selection bias was anticipated.
Ethics oversight	The study was approved by the Ethics Institutional Review Boards of the First Affiliated Hospital of Sun Yat-sen University (No. [2020]322) and complied with all relevant ethical regulations regarding human participants.

Note that full information on the approval of the study protocol must also be provided in the manuscript.

Field-specific reporting

Please select the one below that is the best fit for your research. If you are not sure, read the appropriate sections before making your selection.

☒ Life sciences ☐ Behavioural & social sciences ☐ Ecological, evolutionary & environmental sciences

For a reference copy of the document with all sections, see nature.com/documents/nr-reporting-summary-flat.pdf

Life sciences study design

All studies must disclose on these points even when the disclosure is negative.

Sample size	For animal studies, sample size was chosen to comply with the 3R principles to minimize the number of mice used. For clinical data, sample size was determined based on our previous experience (PMID: 33664496 and 35970825) and the number of samples with available prognostic information.
Data exclusions	Animals failing to grow tumour after intracranial injection of glioma stem cells were excluded.
Replication	All data were reliably reproduced. The number of repeats is indicated in each figure legend.
Randomization	For in vivo studies, all mice were randomly divided into each group without any self-selection before the start of each experiment.<br>For clinical data analysis, all glioma samples were collected from patients who received no treatment before surgery. They were allocated to high- or low-expression group according to the expression of MET404 determined by semiquantitative western blot. Covariates such as sex, age were controlled by ensuring comparable proportion of patient with different age or sex were allocated to each group. Extra cases were excluded, and final cohort consisted of 71 patients in the prognosis analysis.
Blinding	Investigators were not blinded to most animal experiments as interventions required knowledge for individual experimental groups. To ensure overall survival experiments were performed in the blinded manner, multiple co-authors were involved in the assessment of the disease symptoms. Postmortem analysis was performed in a blinded way.

Reporting for specific materials, systems and methods

We require information from authors about some types of materials, experimental systems and methods used in many studies. Here, indicate whether each material, system or method listed is relevant to your study. If you are not sure if a list item applies to your research, read the appropriate section before selecting a response.

Materials & experimental systems

n/a	Involved in the study
☐	☒ Antibodies
☐	☒ Eukaryotic cell lines
☒	☐ Palaeontology and archaeology
☐	☒ Animals and other organisms
☒	☐ Clinical data
☒	☐ Dual use research of concern

Methods

n/a	Involved in the study
☒	☐ ChIP-seq
☐	☒ Flow cytometry
☒	☐ MRI-based neuroimaging

Antibodies

Antibodies used

anti-m6A antibody (Synaptic Systems, 202003)
anti-MET404 (GenScript custom antibody, clone 1C11)
anti-ALKBH5 (Proteintech, 16837-1-AP)
anti-YTHDF2 (Proteintech, 24744-1-AP)
anti-YTHDF1 (Proteintech, 17479-1-AP)
anti-YTHDF3 (Proteintech, 25537-1-AP)
anti-P53 (Proteintech, 60283-2-Ig, clone 6C4B6)
anti-MET (N-terminus specific, Abcam, ab51067, clone EP1454Y)
anti-MET (Cell Signaling Technology, 8198, clone D1C2)
anti-phospho-MET Tyr1234/1235 (Cell Signaling Technology, 3077, clone D26)
anti-AKT (Cell Signaling Technology, 4691, clone C67E7)
anti-phospho-AKT Ser473 (Cell Signaling Technology, 4060, clone D9E)
anti-ERK1/2 (SAB, 29935)
anti-phospho-ERK1/2 (SAB, 52094, clone S05-2H9)
anti-EGFR (Boster, A00023-4)
anti-phospho EGFR Y1068 (Abcam, ab40815, clone EP774Y)
anti-PDGFR alpha (Abcam, ab248689, clone EPR5480)
anti-phospho PDGFR alpha Y720 (Abcam, ab134068, clone EP2478)
anti-FGFR1 (Abcam, ab76464, clone EPR806Y)
anti-phospho FGFR1 Y654 (Abcam, ab59194)
anti-panTrk (Abcam, ab76291, clone EP1058Y)
anti-phospho TrkB Y705 (Abcam, ab229908, clone EPR22298-67)
anti-METTL3 (Proteintech, 15073-1-AP)
anti-6x-His tag (Abcam, 18184, clone HIS.H8)
anti-flag tag (F1804, Sigma-Aldrich, clone M2)
anti-HA tag (Sigma-Aldrich, H6908)
anti-β-actin (Sigma-Aldrich, A1978, clone AC-15)
anti-β-tubulin (Cell Signaling Technology, 2128, clone 9F3)
anti-GFAP (Abcam, ab7260)
anti-Nestin (Abcam, ab221660, clone EPR22023)
anti-Ki67 (Abcam, ab15580)
anti-CD133-PE (566593, BD Biosciences, clone W6B3C1)
HRP-conjugated secondary antibodies (5220-0336/5220-0341, SeraCare)
Antibody dilution is specified in the methods section of the manuscript.

Validation

The anti-MET404 antibody specifically targets human MET404 C-terminus INLS. It was validated in this paper with knock-down cell lines as control in immunoblotting experiments (Fig. 2f and Fig. 3b). Uncropped full scan blots of using this antibody to detect purified MET404 and endogenous MET404 in cell lysates were provided to validate its specificity (Supplementary Fig. 3e).

Other antibodies have been validated by manufacturers, with detailed specificity described on their websites:
anti-m6A antibody (Synaptic Systems, 202003): Specific for N6-methyladenosine (m6A), validated for dot plot, IP. [https://www.sysy.com/product/202003#list]
anti-ALKBH5 (Proteintech, 16837-1-AP): Recognizing human and mouse ALKBH5, validated for IHC, WB, ELISA. [https://www.ptgcn.com/products/ALKBH5-Antibody-16837-1-AP.htm]
anti-YTHDF2 (Proteintech, 24744-1-AP): Recognizing human and mouse YTHDF2, validated for IF, IP, WB, ELISA. [https://www.ptgcn.com/products/YTHDF2-Antibody-24744-1-AP.htm]
anti-YTHDF1 (Proteintech, 17479-1-AP): Recognizing human and mouse YTHDF1, validated for IF, IHC, IP, WB, ELISA. [https://www.ptgcn.com/products/YTHDF1-Antibody-17479-1-AP.htm]
anti-YTHDF3 (Proteintech, 25537-1-AP): Recognizing human and mouse YTHDF3, validated for IF, WB, ELISA. [https://www.ptgcn.com/products/YTHDF3-Antibody-25537-1-AP.htm]
anti-P53 (Proteintech, 60283-2-Ig): Recognizing human and mouse P53, validated for IF, IHC, WB, ELISA. [https://www.ptgcn.com/products/P53-Antibody-60283-2-Ig.htm]
anti-MET (N-terminus specific, Abcam, ab51067): Recognizing human and mouse MET, validated for WB, IHC, ELISA. [https://www.abcam.com/products/primary-antibodies/met-c-met-antibody-ep1454y-n-terminal-ab51067.html]
anti-MET (Cell Signaling Technology, 8198): Recognizing human MET, validated for WB, IHC, IF, IP. [https://www.cellsignal.cn/products/primary-antibodies/met-d1c2-xp-rabbit-mab/8198?site-search-type=Products&N=4294956287&Ntt=8198&fromPage=plp&_requestid=3491109]
anti-phospho-MET Tyr1234/1235 (Cell Signaling Technology, 3077): detects endogenous levels of Met only when phosphorylated at Tyr1234/1235 in human and mouse, validated for WB, IP, IHC, IF. [https://www.cellsignal.com/products/primary-antibodies/

phospho-met-tyr1234-1235-d26-xp-rabbit-mab/3077?site-search-type=Products&N=4294956287&Ntt=3077&fromPage=plp&_requestid=3491586&country=USA]
anti-AKT (Cell Signaling Technology, 4691): detects endogenous levels of total Akt protein in human and mouse, validated for WB, IP, IHC, IF. [https://www.cellsignal.com/products/primary-antibodies/akt-pan-c67e7-rabbit-mab/4691]
anti-phospho-AKT Ser473 (Cell Signaling Technology, 4060): detects Akt phosphorylated at Ser473 in human and mouse. , validated for WB, IP, IHC, IF. [https://www.cellsignal.com/products/primary-antibodies/phospho-akt-ser473-d9e-xp-rabbit-mab/4060]
anti-ERK1/2 (SAB, 29935): Recognizing human and mouse ERK, validated for WB, IHC, IF. [https://www.sabbiotech.com/g-201031-ERK1-ERK2-Polyclonal-Antibody-29935.html]
anti-phospho-ERK1/2 (SAB, 52094): Recognizing human and mouse ERK phosphorylated at Thr202/Thr185, validated for WB. [https://www.sabbiotech.com/g-314073-Phospho-ERK1-2-(Thr202-Thr185)-Rabbit-mAb-52094.html]
anti-EGFR (Boster, A00023-4): Recognizing human and mouse EGFR, validated for ELISA, Flow Cytometry, IF, IHC, ICC, WB. [https://www.bosterbio.com/anti-egfr-picoband-trade-antibody-a00023-4-boster.html]
anti-phospho EGFR Y1068 (Abcam, ab40815): Recognizing EGFR phosphorylated on Tyrosine 1068 in human and mouse, validated for WB, IF, IHC. [https://www.abcam.com/products/primary-antibodies/egfr-phospho-y1068-antibody-ep774y-ab40815.html]
anti-PDGFR alpha (Abcam, ab248689): Recognizing PDGFR alpha in human and rat, validated for IHC, WB, ELISA. [https://www.abcam.com/products/primary-antibodies/pdgfr-alpha-antibody-epr5480-bsa-and-azide-free-ab248689.html]
anti-phospho PDGFR alpha Y720 (Abcam, ab134068): Recognizing PDGFR alpha phosphorylated at Y720 in human and mouse, validated for WB, IF. [https://www.abcam.com/products/primary-antibodies/pdgfr-alpha-phospho-y720-antibody-ep2478-ab134068.html]
anti-FGFR1 (Abcam, ab76464): Recognizing human FGFR1, validated for WB, IP, IF. [https://www.abcam.com/products/primary-antibodies/fgfr1-antibody-epr806y-ab76464.html]
anti-phospho FGFR1 Y654 (Abcam, ab59194): Recognizing human and mouse FGFR1 phosphorylated at Tyr654, validated for WB, IHC, IF. [https://www.abcam.com/products/primary-antibodies/fgfr1-phospho-y654-antibody-ab59194.html]
anti-panTrk (Abcam, ab76291): Recognizing human and mouse pan Trk, validated for ELISA, IF, WB, IP, IHC. [https://www.abcam.com/products/primary-antibodies/pan-trk-antibody-ep1058y-ab76291.html]
anti-phospho TrkB Y705 (Abcam, ab229908): Recognizing human and mouse phosphorylated TrkB Y705, validated for WB. [https://www.abcam.com/products/primary-antibodies/trkb-phospho-y705-antibody-epr22298-67-ab229908.html]
anti-METTL3 (Proteintech, 15073-1-AP): Recognizing human and mouse METTL3, validated for ChIP, IP, IF, WB, RIP. [https://www.ptgcn.com/products/METTL3-Antibody-15073-1-AP.htm]
anti-6x-His tag (Abcam, 18184): Recognizing his-tagged recombinant proteins, validated for IF, WB, IP. [https://www.abcam.com/products/primary-antibodies/6x-his-tag-antibody-hish8-ab18184.html]
anti-flag tag (F1804, Sigma-Aldrich): Recognizing N-Asp-Tyr-Lys-Asp-Asp-Asp-Asp-Lys-C, validated for IF, WB, IP. [https://www.sigmaaldrich.cn/CN/zh/product/sigma/f1804]
anti-HA tag (Sigma-Aldrich, H6908): Recognizing HA-tagged recombinant proteins, validated for WB, IP. [https://www.sigmaaldrich.cn/CN/zh/product/sigma/h6908]
anti-β-actin (Sigma-Aldrich, A1978): Recognizing an epitope located on the N-terminal end of the β-isoform of actin in human and mouse, etc.; validated for WB, IHC, IF [https://www.sigmaaldrich.cn/CN/en/product/sigma/a1978]
anti-β-tubulin (Cell Signaling Technology, 2128): Recognizing human and mouse β-tubulin, validated for WB, IHC, IF. [https://www.cellsignal.cn/products/primary-antibodies/b-tubulin-9f3-rabbit-mab/2128?site-search-type=Products&N=4294956287&Ntt=2128&fromPage=plp&_requestid=3499524]
anti-GFAP (Abcam, ab7260): Recognizing mouse GFAP, validated for IHC, IF, WB. [https://www.abcam.com/products/primary-antibodies/gfap-antibody-ab7260.html]
anti-Nestin (Abcam, ab221660): Recognizing mouse and rat Nestin, validated for WB, IHC. [https://www.abcam.com/products/primary-antibodies/nestin-antibody-epr22023-ab221660.html]
anti-Ki67 (Abcam, ab15580): Recognizing mouse and human Ki-67, validated for IHC, IF. [https://www.abcam.com/products/primary-antibodies/ki67-antibody-ab15580.html]
anti-CD133-PE (566593, BD Biosciences): Recognizing human CD133, validated for flow cytometry. [https://www.bdbiosciences.com/en-sg/products/reagents/flow-cytometry-reagents/research-reagents/single-color-antibodies-ruo/pe-mouse-anti-human-cd133.566593]

Eukaryotic cell lines

Policy information about cell lines and Sex and Gender in Research

Cell line source(s)	Glioma stem cell line, including 387, 456, 28, and 23, were kindly provided by Dr. Jeremy Rich, UPMC.<br>Glioma cell line U251 (09063001, ECACC) and SNB19 (ACC 325, DSMZ) cells were kindly provided by Dr. Suyun Huang, VCU.<br>HEK293T (CRL-11268, ATCC)<br>U118 (HTB-15, ATCC)<br>NHA (CC-2565, Lonza)<br>Neural stem cell (A15654, Thermo Fisher and TM A15654, Gibco)
Authentication	Cells were authenticated using short tandem repeat (STR) fingerprinting where applicable.
Mycoplasma contamination	All cell lines were tested negative for mycoplasma.
Commonly misidentified lines (See ICLAC register)	No commonly misidentified cell lines were used in this study.

Animals and other research organisms

Policy information about studies involving animals; ARRIVE guidelines recommended for reporting animal research, and Sex and Gender in Research

Laboratory animals	6-12 week old circMET knockout mice (C57BL/6 background)

Laboratory animals	0.5-1 year old MET404 knock-in (P53f/f; R26-MET404-flagLSL/+; GFAP-Cre) and control ((P53f/f; R26-MET404-flag+/+; GFAP-Cre)) mice, C57BL/6 background<br>6 week old female BALB/c-nu mice
Wild animals	The study did not involve wild animals.
Reporting on sex	Sex has not been confirmed as a critical factor for glioma tumorigenesis and prognosis. To exclude potential effects from sex, the same number of male and female mice were assigned in each group. Source data disaggregated for sex has been collected and will be provided with the revised manuscript, if applicable.
Field-collected samples	The study did not involve field-collected samples.
Ethics oversight	All experimental protocols concerning the handling of mice were approved by the Ethics Institutional Review Boards of the First Affiliated Hospital of Sun Yat-sen University (No. [2021]059).

Note that full information on the approval of the study protocol must also be provided in the manuscript.

Flow Cytometry

Plots

Confirm that:

☒ The axis labels state the marker and fluorochrome used (e.g. CD4-FITC).

☒ The axis scales are clearly visible. Include numbers along axes only for bottom left plot of group (a 'group' is an analysis of identical markers).

☒ All plots are contour plots with outliers or pseudocolor plots.

☒ A numerical value for number of cells or percentage (with statistics) is provided.

Methodology

Sample preparation	Fresh human glioma tissue was minced, digested (collagenase IV (1 mg/ml, Gibco), DNase I (20 U/ml, Sigma–Aldrich), hyaluronidase (0.01%, Solarbio) and DMEM/F12 (Gibco)) for 40 minutes at 37 °C, and sequentially filtered through 70-µm and 40-µm strainers. Myelin was removed by Debris Removal Solution (130-109-398, Miltenyi Biotec) according to the manufacturer's instructions. Red blood cells were then lysed with RBC lysis buffer (C3702, Beyotime). Then, the collected cell pellet was resuspended in FACS staining buffer (PBS containing 2% FBS).
Instrument	BD FACSAriaII
Software	FACS Diva software (version 7)
Cell population abundance	Post-sort fractions were >95% as determined by a post-sort purity check of representative samples.
Gating strategy	Gating strategy is provided in Supplementary Figure 4, with dead cells and non-singlets excluded.<br>Gating for glioma stem cell: live single cells, CD133+

☒ Tick this box to confirm that a figure exemplifying the gating strategy is provided in the Supplementary Information.
